# Efficient NiFe-Layered Double Hydroxide Electrocatalyst Synthesized via a Solvent-Free Mechanochemical Method for Oxygen Evolution Reaction

**DOI:** 10.1021/acsomega.4c11115

**Published:** 2025-05-30

**Authors:** Manuel Molina-Muriel, Sabrina Campagna Zignani, Sara Goberna-Ferrón, Antonio Ribera, Antonino Salvatore Aricò, Hermenegildo García

**Affiliations:** † Instituto de Tecnología Química CSIC-UPV, 16774 Universitat Politècnica de València and Consejo Superior de Investigaciones Científicas, Universitat Politècnica de València, Av. de los Naranjos s/n, Valencia 46022, Spain; ‡ Institute of Advanced Energy Technologies (ITAE) of the Italian National Research Council (CNR), Via Salita S. Lucia sopra Contesse 5, Messina 98126, Italy; § Departamento de Química Inorgánica, Universitat de València, Carrer del Doctor Moliner, 50 Burjassot, Valencia 46100, Spain

## Abstract

The growing concern over climate change and the reliance on fossil fuels has spurred interest in alternative energy processes, particularly electrochemical water splitting to produce hydrogen (H_2_). This study focuses on developing cost-effective and efficient oxygen evolution reaction (OER) electrocatalysts. We report a novel solvent-free mechanochemical method for synthesizing NiFe-layered double hydroxide (LDH), which demonstrates promising electrocatalytic properties for the OER. The mechanochemical synthesis, requiring only 1 h of solid reagent grinding, produces NiFe-LDH with structural features comparable to those obtained via traditional aqueous phase methods. The electrocatalyst was evaluated in a single cell with a membrane-electrode assembly configuration under alkaline conditions, exhibiting an overpotential of 221 mV at a current density of 10 mA·cm^–2^ and a Tafel slope of 103.1 mV·dec^–1^, indicating excellent OER kinetics and low energy barriers. Additionally, the catalyst demonstrated robust durability, maintaining a potential of around 1.55 V during a 35 h test at high current densities of 0.1 A·cm^–2^ and even 1.75 V at 1 A·cm^–2^. This work highlights the potential of NiFe-LDH synthesized by an energy-efficient, environmentally green, and scalable process for large industrial water-splitting applications, contributing to the advancement of sustainable hydrogen production technologies.

## Graphical abstract

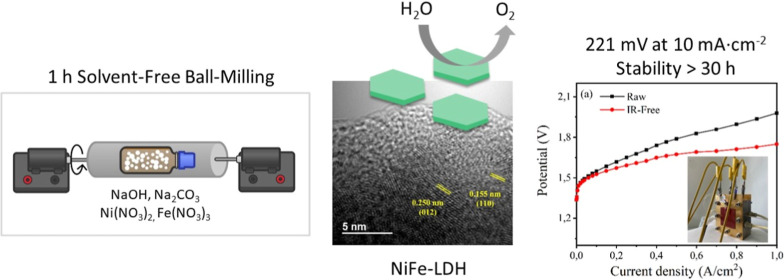

## 1 - Introduction

Recent years have seen growing awareness of climate change due to fossil fuel usage, leading to increased interest in alternative energy processes.^1–4^ Electrochemical water splitting to produce H_2_ is considered as one of the most desirable and feasible methods for clean energy production.^5–7^ However, the sluggish kinetics of oxygen evolution reaction (OER), which involves the transfer of four electrons, four protons and O–O bond formation, hampers the efficient performance of these systems.^8–10^ Thus, developing electrocatalysts with high OER efficiency is crucial for practical and efficient overall water splitting.

Noble metals and their derivatives are commonly used as OER electrocatalysts, but their use is limited by high costs and low availability.^11–13^ Therefore, developing cost-effective alternatives with similar activity based on abundant elements is essential.^14,15^ In this regard, transition-metal oxides, phosphides, perovskites, and carbon-based materials have shown satisfactory performance as OER electrocatalysts.^16–19^

Layered double hydroxides (LDH), particularly bimetallic systems containing Ni and Fe, stand out due to their good performance, tunability, stability, and low environmental impact.^15,20–23^ Despite the advantages of transition metal-based LDHs, their synthetic procedures often require high temperatures or the use of solvents, increasing production costs, and resulting in considerable volumes of aqueous wastes.^24,25^

Since Tongamp et al. described in 2007 the solvent-free mechanochemical synthesis of a LDH material for the first time, this kind of synthetic procedure has emerged as a cost-efficient procedure for the preparation of LDH-based materials with a minimal energy investment and waste generation.^26,27^ Following the first findings on this field, more studies related to the application of mechanochemically synthesized LDH have been published, including some in which the prepared materials were used as suitable OER electrocatalysts.^28–33^

Herein, in this work, an efficient NiFe-LDH electrocatalyst was prepared using a solvent-free mechanochemical synthetic procedure, showing structural features similar to those of LDH synthesized by conventional methods. The activity of the prepared material for electrocatalytic OER was evaluated in a single electrocatalytic cell with a membrane-electrode assembly (MEA) configuration under alkaline conditions, demonstrating good performance and stability during a 30 h durability test at high current density values required for industrial applications.

## 2 Materials and Methods

### 2.1 Materials

Ni­(NO_3_)_2_·6H_2_O, Fe­(NO_3_)_3_·9H_2_O, NaOH, KOH, and Na_2_CO_3_ were purchased from Sigma-Aldrich. Platinum nominally 40% on carbon black was obtained from Alfa Aesar. Water used in all experiments was Milli-Q purity grade.

### 2.2 Catalyst Preparation

NiFe-layered double hydroxide (NiFe-LDH) was prepared by a mechanochemical method. In brief, 6.40 g (160 mmol) of NaOH, 1.06 g (10 mmol) of Na_2_CO_3_, and 165 g of ZrO_2_ milling balls (2 mm diameter) were introduced in a 100 mL plastic bottle. The bottle was then placed inside a tubular ball mill, and the reagents were grinded at a speed of 500 rpm. After this step, 17.45 g (60 mmol) of Ni­(NO_3_)_2_·6H_2_O and 8.08 g (20 mmol) of Fe­(NO_3_)_3_·9H_2_O were also introduced in the bottle, and all the components were milled for an additional hour.

After the milling, ZrO_2_ balls were separated from the resulting brown solid by washing with a minimal amount of water (10 mL) and subsequent filtration. The suspension generated was then filtered with a Büchner under vacuum and washed with some additional water (10 mL) to recover the solid, which was dried afterward at room temperature.

For comparison, a NiFe oxohydroxide electrocatalyst was prepared by a traditional coprecipitation method already reported by our group,^34^ using Ni­(NO_3_)_2_·6H_2_O and Fe­(NO_3_)_3_·9H_2_O. The precursors in the desired atomic ratio (85:15 atomic %) were dissolved in Milli-Q water to form a suspension. A NaOH 0.5 M solution was added dropwise until reaching a pH of 9 while maintaining the temperature at 60 °C. The solution was further stirred until precipitation of the amorphous hydroxide. The suspension was filtered, dried, and treated at 120 °C.

### 2.3 Materials Characterization

Powder X-ray diffraction (PXRD) spectra were measured on a Philips X’PERT diffractometer equipped with a graphite monochromator operating at 40 kV and 45 mA and using Cu Kα radiation (λ = 1.54178 Å) in the 2θ range of 5–90 °. Field emission scanning microscopy (FESEM) images were obtained on a ZEISS Ultra 55 apparatus. High-resolution transmission electron microscopy (HRTEM) and high-angle annular dark-field scanning transmission microscopy images (HAADF-STEM), as well as the elemental distribution, were obtained on a JEOL JEM 2100F microscope equipped with an EDS detector. Inductively coupled plasma optical emission spectroscopy (ICP-OES) analysis of the samples was performed on Varian 715 ES equipment. The specific surface area of the material was determined by measuring N_2_ adsorption isotherms at 77.3 K on a Micromeritics ASAP 2010 instrument. X-ray photoelectron spectroscopy analysis was performed on a SPECS spectrometer equipped with a PHOIBOS 150 MCD-9 detector using a nonmonochromatic light source (Al Kα) operating at 200 W. The obtained data were calibrated against the adventitious carbon C 1s at 284.6 eV.

### 2.4 Electrode Preparation

Electrodes were prepared by deposition of the corresponding catalyst suspension in n-propanol over a 5 cm^2^ piece of the corresponding support. For anode preparation, 67 mg of NiFe-LDH was suspended in 15 mL of n-propanol and sonicated for 15 min. Then, a certain amount of ionomer solution (33 wt % of catalyst) was added, and the suspension was sonicated again for another 15 min. Deposition was carried out by spray-coating of the suspension over a Ni-felt support (5 cm^2^) in successive layers, drying the material between each spraying step to increase the adherence. The support was placed on a heating plate at 60 °C during deposition to favor solvent evaporation and adherence of the catalyst. Ionomer solution was prepared by dispersing the Fumasep FAA3-50 membrane in a certain amount of an ethanol:n-propanol mixture (1:1 vol.), with a concentration of 5% wt.

The cathode was prepared by a similar procedure, using a suspension of Pt/C as catalyst, *n*-propanol, and ionomer and a 5 cm^2^ carbon-based Sigracet 25-BC gas diffusion layer (SGL) as support.

### 2.5 Electrochemical Measurements

The prepared electrode was evaluated in a single cell housing constituted by a nickel plate with a geometrical active area of 5 cm^2^. Anodes and cathodes were separated by a polymeric anion exchange membrane (Fumasep FAA3–50).^35^ A schematic representation of the cell is presented in Scheme 1.

**Scheme 1:**
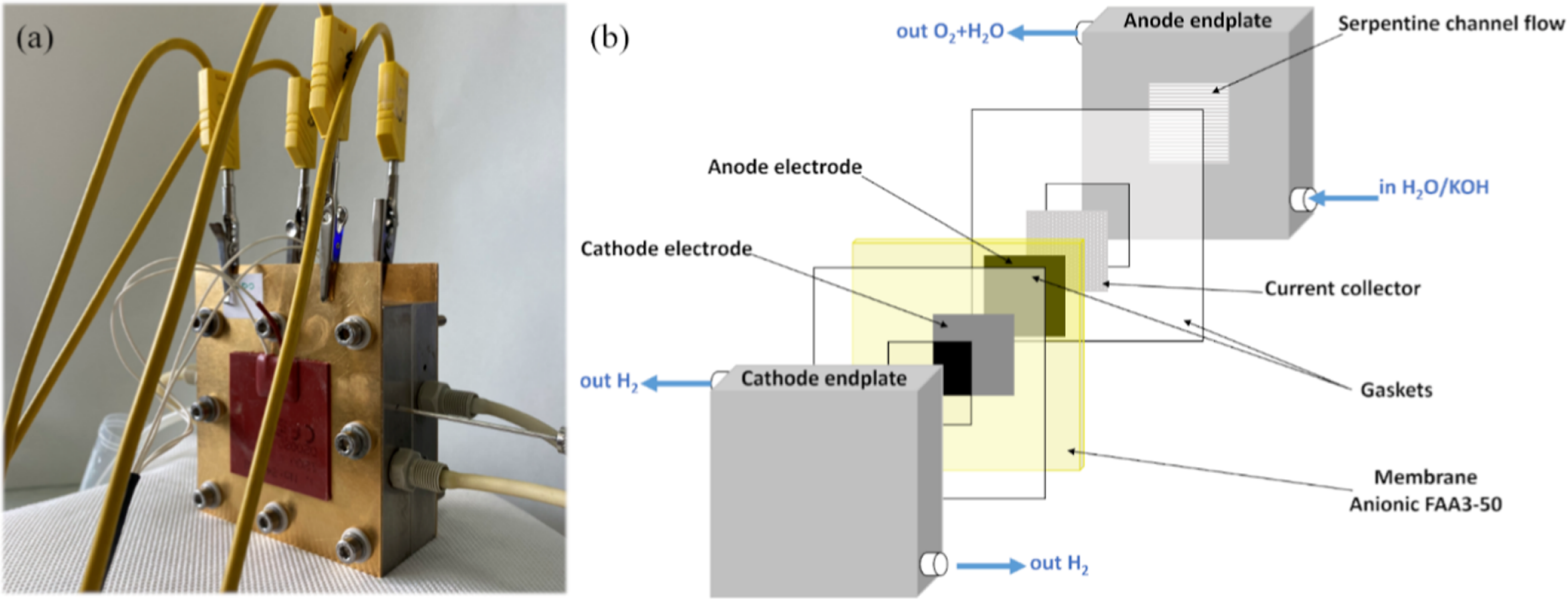
Picture (a) and Schematic (b) Representation of the Used Single Cell

The cell had independent recirculation circuits for the anode and cathode. In the anode, a 1 M KOH solution was recirculated by a pump (4 mL/min) as the electrolyte during the test. Cell temperature was kept at 50 °C during all experiments, controlled by a thermocouple. Prior to the assembly of the electrochemical cell, anion exchange of both electrodes and the membrane by OH^–^ ions was performed by immersion in a 1 M KOH solution during 24 h.

After cell assembly, conditioning of the system was carried out, applying a current of 0.25 A for 24 h. Polarization curves and a durability test were measured with Keithley power supply equipment (Tektronic). Electrochemical impedance spectroscopy (EIS) measurements were conducted in an Autolab PGSTAT302 N potentiostat, equipped with a booster of 20 A (Metrohm) and a frequency response analyzer, in the frequency range from 100 MHz to 10 kHz, at different voltages (1.5, 1.8, and 2 V; 0.01 Vrms) to compare the different performances. Data fitting for the extraction of the elements of the equivalent electrical circuit was carried out by using NOVA software from Metrohm.

## 3 Results and Discussion

### 3.1 Synthesis and Characterization

The novel solvent-free mechanochemical method for NiFe-LDH preparation is illustrated in Scheme 2. First, sodium hydroxide and sodium carbonate were mechanically ground for 5 min, then a designated amount of hydrated nickel and iron nitrates was added, and the mixture was further ground for 1 h at 500 rpm. By this synthetic method, just 1 h of reagent grinding is enough to produce a material with the same structure and features as similar catalysts obtained by traditional aqueous phase methods, like hydrothermal synthesis or coprecipitation. This mechanochemical synthesis method offers several advantages: (i) cost reduction derived from the absence of any solvent and heating process, (ii) easy scaling-up of the process since a large amount of material can be synthesized in a short time, and (iii) minimal water consumption in the washing steps.

**Scheme 2:**
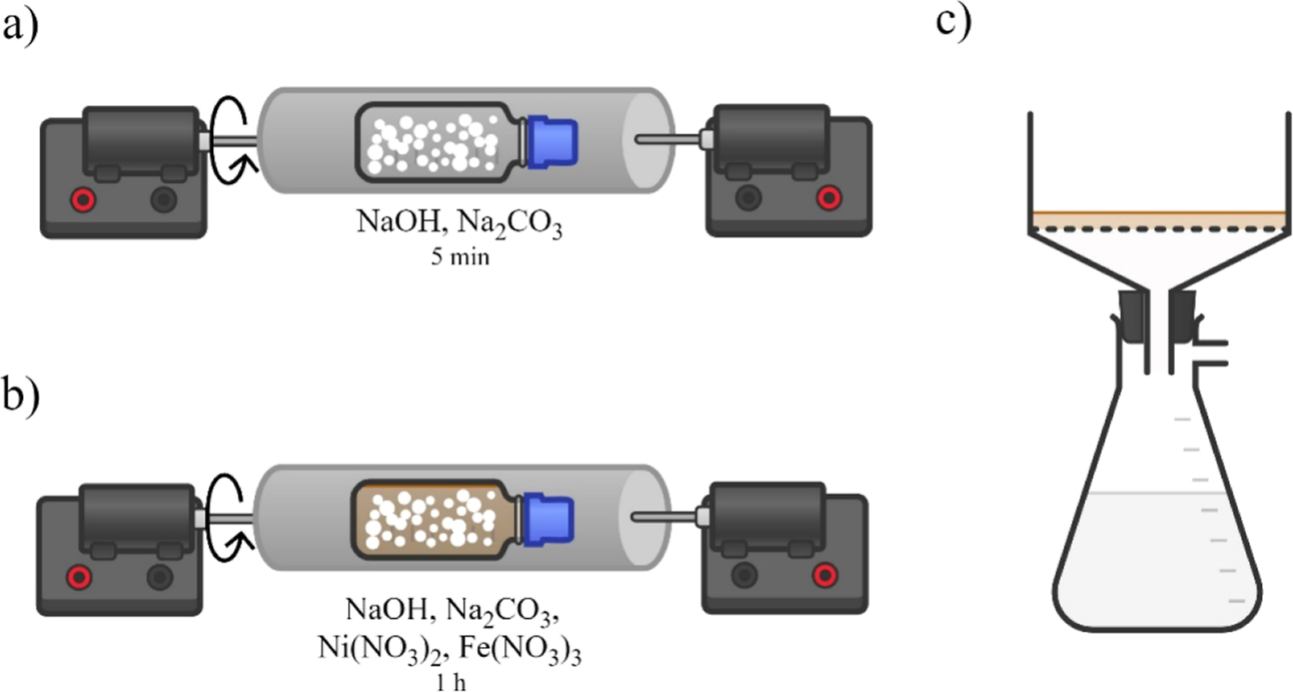
Illustration of the Mechanochemical Synthesis of NiFe-LDH: (a) Grinding of NaOH and Na_2_CO_3_; (b) Grinding after Adding the Metal Precursors; (c) Washing with the Minimal Water Amount and Filtering of the Obtained Solid

PXRD analysis of the material shows the characteristic features of Ni-based LDH with reflections corresponding to (00L) facets at 2θ values of 10.5° (003) and 22.1° (006) and also the (110) plane at a 2θ value of 60.2°. These XRD data are in good agreement with the reported NiFe-LDH XRD data (JCPDS card no. 38-0715),^36^ as seen in Figure 1. The broadness of the PXRD peaks indicates a low degree of crystallinity in the material,^37^ as confirmed by electron microscopy images (Figure 1b–d). The synthesis conditions, including the absence of solvent and external heating, and the short synthesis time hinder particle growth, resulting in a small crystallite size.

**Figure 1:**
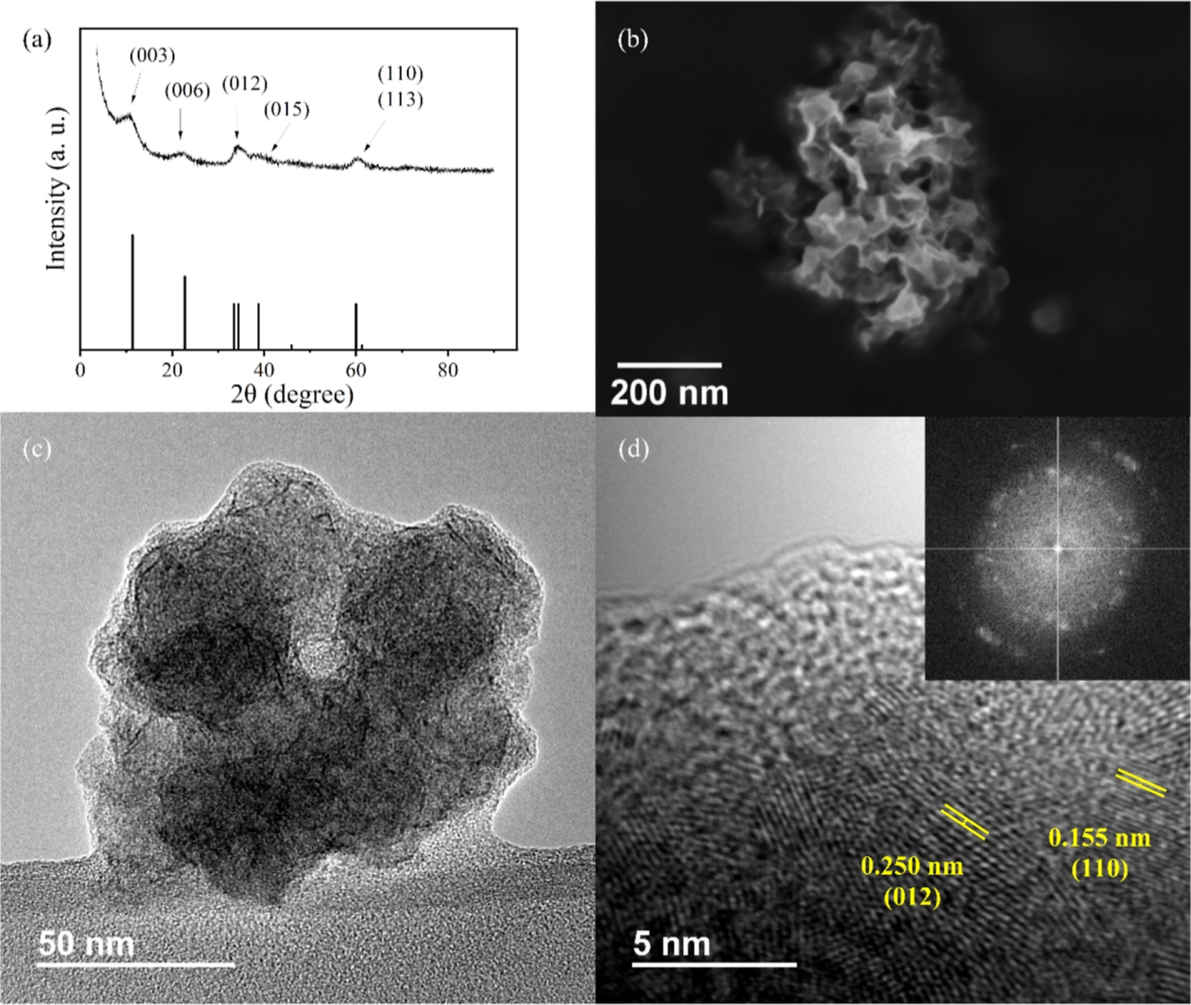
PXRD pattern (a), FESEM (b), TEM (c), and HRTEM (d) images of NiFe-LDH. The inset in (d) shows the SAED pattern of the material.

The metal ratio in the material was determined by ICP-OES. As shown in Table 1, the ratios correspond to a 2.5:1 LDH structure, close to the ratio of precursors added to the mill. The Brunauer–Emmett–Teller surface area was estimated by measuring N_2_ isotherms at 77.3 K. The results are also presented in Table 1.

**Table 1:** Metal Ratios Obtained by ICP-OES and BET Surface Area of NiFe-LDH

	NiFe-LDH
Ni %	71.5
Fe %	28.5
cation ratio	2.5:1
BET area (m^2^/g)	50.2

To identify the morphology of the prepared catalyst, SEM (Figure 1b) images of the sample were obtained. The crystallinity of the material was observed by TEM (Figure 1c,d). As can be seen in Figure 1, the material is composed of an agglomeration of sheet-like particles typical of LDH systems. It can be observed that the size of these agglomerates is around 200 nm, smaller than those prepared by conventional methods,^38^ as it was pointed before.

The crystallographic domains within these agglomerates range from 5 to 10 nm, as indicated by the width at half peak height of the X-ray diffraction peaks. The sheet morphology is more evident in the TEM images. In Figure 1c, it can be clearly seen that particles are composed by the aggregation of nanosheets in the range of tens of nanometers. The high-resolution image in Figure 1d shows the typical spacing of LDH structures that can be assigned to (012) and (110) crystallographic planes, in concordance with PXRD data. In addition, the homogeneous distribution of the constituting elements was confirmed by EDS elemental mapping over the HAADF-STEM images of the material, as seen in Figure S1.

The elemental composition and the oxidation state of the elements on the surface of the material were studied by XPS, and the results are shown in Figure 2. The survey XPS spectrum confirms the presence of Ni, Fe, and O. The Ni 2p high-resolution spectrum shows the characteristic features of Ni^2+39^, with components assigned to Ni 2p_3/2_ at 858.8 eV and Ni 2p_1/2_ at 876.4 eV, accompanied by their respective satellite peaks. The low signal-to-noise ratio in Fe high-resolution XPS peaks is due to the lower concentration of this element on the material, compared to Ni or O. This Fe 2p spectrum contains two components, at 716.1 and 727.7 eV, assigned to Fe 2p_3/2_ and Fe 2p_1/2_ levels, respectively.^39^ The O 1s high-resolution XPS peak can be deconvoluted into one major component at 534.5 eV related to oxygen atoms in hydroxide groups, and two minor components at 532.9 and 536.9 eV, assigned to oxide and adsorbed water, respectively.^40^

**Figure 2:**
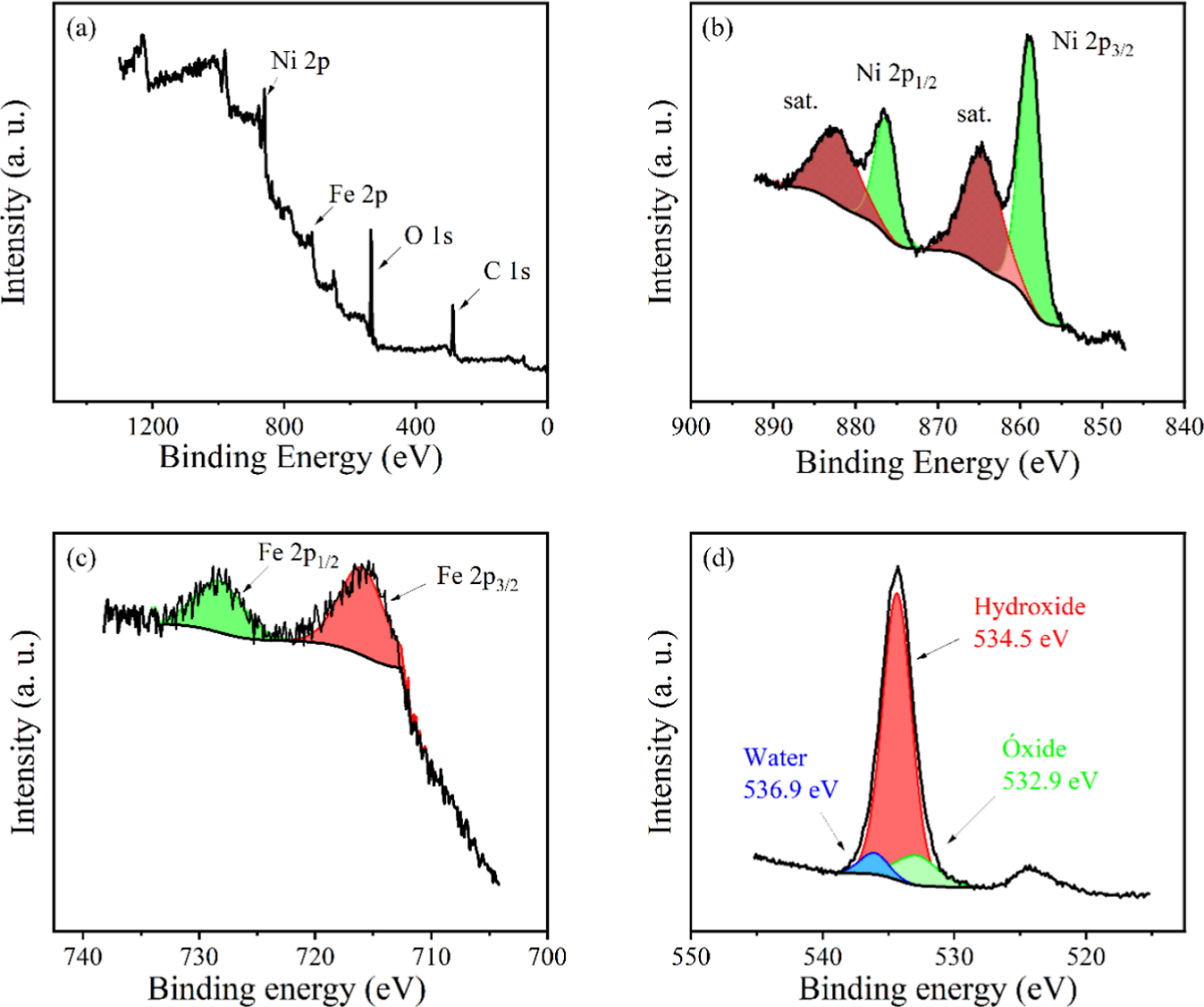
XPS survey spectra of NiFe-LDH (a). High-resolution XPS Ni 2p (b), Fe 2p (c), and O 1s (d) peaks of NiFe-LDH.

### 3.2 Electrochemical Measurements

The electrocatalytic performance of NiFe-LDH was assessed in a MEA configuration using a single-cell setup (Scheme 1). The anode comprised the synthesized NiFe-LDH catalyst (2 mg·cm^–2^), while the cathode used Pt/C (1 mg·cm^–2^) as the catalyst, with an anion exchange membrane (FAA3-50) separating the electrodes. A 1 M KOH solution was recirculated at 4 mL/min, and the cell temperature was maintained at 50 °C throughout the measurements.

Figure 3a presents polarization curves recorded after prolonged conditioning of the MEA. The NiFe-LDH anode exhibited an overpotential of 221 mV at a current density of 10 mA·cm^–2^, alongside a Tafel slope of 103.1 mV·dec^–1^ (Figure 3b). Table 2 compares the electrocatalytic performance of NiFe-LDH synthesized via the mechanochemical method with other NiFe-based catalysts for the OER. The mechanochemically synthesized NiFe-LDH shows a competitive overpotential (221 mV), although the Tafel slope (103 mV·dec^–1^) is higher than those of some advanced catalysts. Nonetheless, the simplicity, scalability, and cost-efficiency of the mechanochemical process make it a promising candidate for large-scale, sustainable hydrogen production. Additionally, a direct comparison with a NiFe OER electrocatalyst synthesized via traditional coprecipitation, evaluated under the same cell configuration, revealed a comparable performance to the mechanochemically synthesized NiFe-LDH (Figure S2 and Table S1).

**Figure 3:**
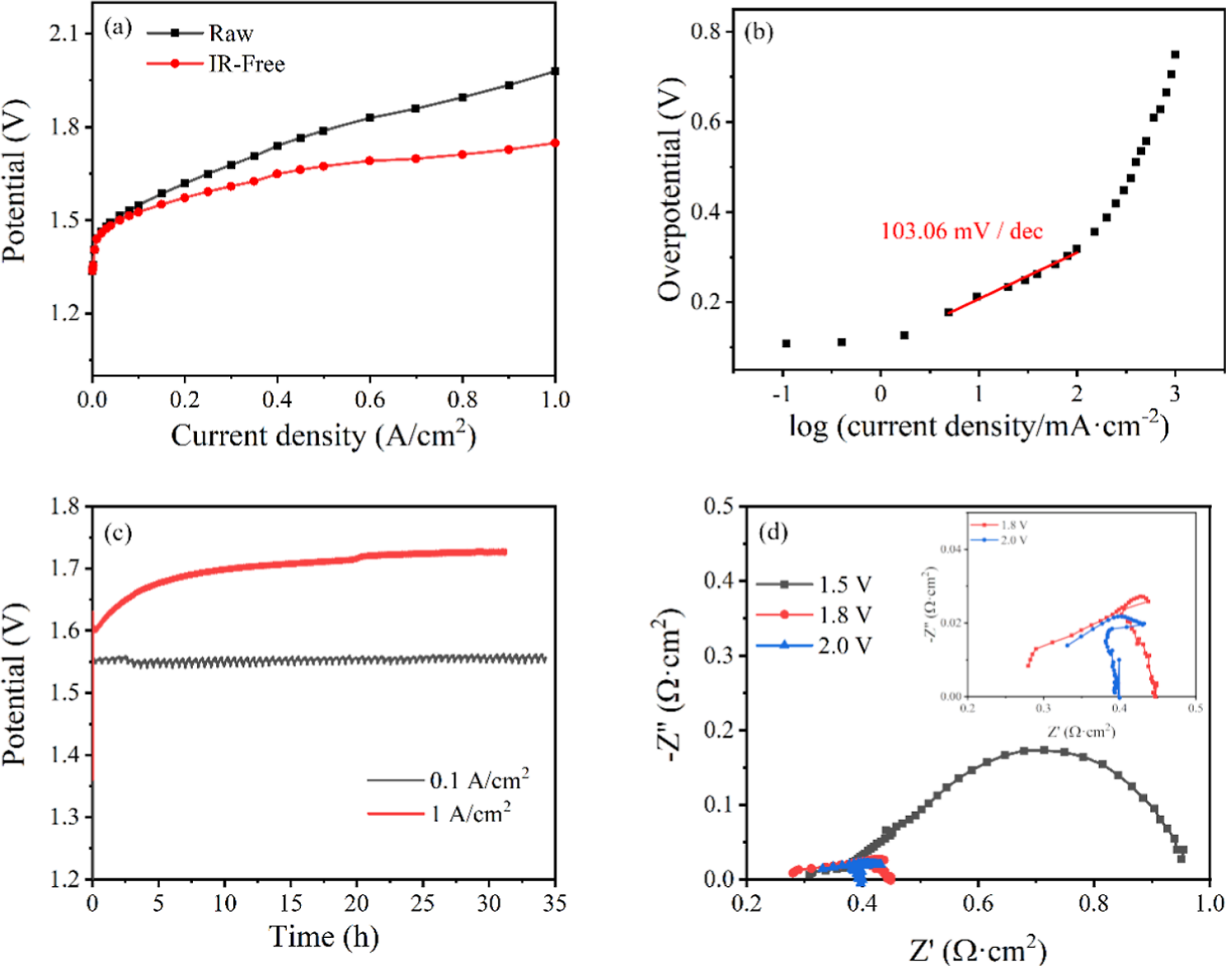
Polarization curves (a), Tafel plot (b), durability test (c), and EIS plot at different applied voltages (d) for NiFe-LDH. The inset in (d) shows a more detailed look of the EIS data obtained at 1.8 and 2.0 V.

**Table 2:** Comparison of Mechanochemical NiFe-LDH Performance with Some NiFe Electrocatalysts Reported in the Literature for OER

catalyst	synthesis method	water used (ml H_2_O/mmol metals)^a^	η at 10 mA·cm^–2^ (mV)	Tafel slope (mV/dec)	ref
vacancy-rich NiFe-LDH@NF	hydrothermal	25	184	57	^15^
Ni_2_P@NiFe hydroxide	hydrothermal followed by thermal treatment	40	205	32	^20^
NiFe(OH)_*x*_/(Ni, Fe)Se_2_	hydrothermal	11	180	42	^21^
NiFe-LDH	mechanochemical	/^b^	280	116	^33^
amorphous NiFe oxide	coprecipitation	167	228	37	^37^
NiFe-LDH hollow microspheres	hydrothermal	9	290	51	^41^
NiFe-LDH	coprecipitation	13	249	24	^42^
NiFe nanoparticles on graphene	coprecipitation followed by freeze-drying	650	292	48	^43^
NiFe alloy@NiFe oxide	atomization and reduction with H_2_		340	49	^44^
NiFe-LDH/TA2	hydrothermal	13	249	62	^45^
NiFe-LDH	mechanochemical	0.25^b^	221	103	this work

a The amount of water or solvents used for washing the catalysts are not included.

b Including water used for washing the catalyst.

The stability of the NiFe-LDH electrocatalyst was confirmed through a 35 h durability test (Figure 3c), maintaining a stable potential of around 1.55 V at 0.1 A·cm^–2^. These results underscore the catalyst’s robustness, making it suitable for long-term use in OER applications. Even at much higher current densities of 1 A·cm^–2^, the potential becomes stabilized at 1.75 V. To further assess the structural and chemical stability of the catalyst, XPS analysis was performed after the stability test (Figure S4). The resulting spectra show no significant differences compared to the fresh sample (Figure 2), suggesting that NiFe-LDH maintains its chemical composition and oxidation states under prolonged electrochemical operation.

AC impedance spectroscopy (EIS) measurements of the NiFe-LDH anode electrode are also presented in Figure 3, conducted at the beginning of experiments and at different cell voltages. At cell voltages between 1.8 and 2 V, the impedance spectra revealed a low-frequency resistance (Rp) of approximately 0.4 Ω·cm^2^, indicating efficient charge transfer and minimal energy loss during the OER. Additionally, the polarization resistance was less than 0.2 Ω·cm^2^, further underscoring the catalyst’s effectiveness in facilitating the OER. The series resistance, around 0.28 Ω·cm^2^, reflected the intrinsic resistance of the electrolyte and other cell components. These values are notably favorable when compared to those at 1.5 V, where the low-frequency resistance was 0.95 Ω·cm^2^ and the polarization resistance was 0.65 Ω·cm^2^. The polarization resistance was determined as the difference between the low- and high-frequency intercepts on the real axis. This behavior, with a strong performance across the 1.5–2 V range, highlights the robustness and efficiency of the NiFe-LDH electrocatalyst. Using this information, the equivalent circuit model of the system was proposed, as shown in Figure S3. The parameters of the equivalent circuit model are given in Table S2.

The achieved performances are in line with the state of the art of NiFe-LDH electrocatalysts for alkaline water electrolysis, with the specific advantage of an effective and easy-scale-up preparation procedure. This catalyst can also be applied in parallel technologies such as alkaline electrolysis and CO_2_-water electrolysis. However, in comparison with the conventional aqueous phase LDH synthesis, the present material consumes about 2 orders of magnitude less water and, therefore, produces much fewer waste waters that are necessary to be treated. The reasoning for this is the absence of solvent during the synthesis process and the use of stoichiometric amounts of precursors and nitrate salts (highly soluble in water), which reduces the amount of water required for washing the material. For instance, in the synthesis of NiFe­(OH)_x_/(Ni, Fe)­Se_2_, the best-performing NiFe material in Table 2, over 40-fold higher water volume is needed in the synthesis, plus an undetermined volume of water for washing the material, thus diminishing its practicality in large-scale production.

## 4 Conclusions

In the present work, a NiFe-LDH OER electrocatalyst was prepared by a novel, solvent-free mechanochemical method. By this procedure, just mechanical grinding of the precursors for 1 h at ambient temperature is sufficient to obtain a material with the characteristic feature of LDH prepared by traditional synthetic methods. The short synthesis time and absence of external heating result in a material with small particle size, as confirmed by PXRD data and electron microscopy images. This low degree of crystallinity and small particle size are beneficial for its electrocatalytic OER performance.

The promising electrocatalytic performance, stability, and cost-effectiveness of NiFe-LDH synthesized via mechanochemical methods highlight its potential for practical water electrolysis applications. Unlike many studies that rely on basic H-cells for electrochemical characterization, this work employs an MEA configuration, a setup much closer to real industrial applications. The low overpotential of 221 mV at 10 mA·cm^–2^, competitive Tafel slope of 103.1 mV·dec^–1^, and extended durability under high current densities (up to 1 A·cm^2^) further reinforce the feasibility of NiFe-LDH as an anode catalyst for the OER in alkaline water electrolysis. Additionally, the solvent-free synthesis method significantly reduces energy consumption and waste generation, consuming up to 40 times less water than conventional hydrothermal or coprecipitation methods. These attributes position NiFe-LDH as a strong candidate for integration into commercial alkaline water electrolyzers, contributing to the advancement of sustainable hydrogen generation technologies. Furthermore, the versatile nature of the mechanochemical synthesis described in this work opens avenues for its adaptation to the preparation of other electrocatalysts, potentially extending its applicability to various energy conversion and storage technologies.

## Supplementary Material



## Notes

HAADF-STEM image and elemental distribution of NiFe-LDH; comparative polarization curves and Tafel slopes for NiFe-LDH and NiFe oxohydroxide; overpotential and Tafel slope values for NiFe-LDH and NiFe oxohydroxide; proposed equivalent circuit model for the electrocatalytic system; parameters obtained for the equivalent circuit model; and high-resolution XPS spectra of Ni 2p, Fe 2p, and O 1s after 35 h durability test (PDF)
