# Supplementary material for: Efficient NiFe-Layered Double Hydroxide Electrocatalyst Synthesized via a Solvent-Free Mechanochemical Method for Oxygen Evolution Reaction
Source: ACS Omega. 2025 May 30;10(22):22671–8. doi: 10.1021/acsomega.4c11115 (PMC12163631; doi:10.1021/acsomega.4c11115)
Supplement: Supplementary file 1 [file ao4c11115_si_001.pdf]

Supporting information

Efficient NiFe-Layered Double Hydroxide Electrocatalyst Synthesized via Solvent-Free Mechanochemical Method for Oxygen Evolution Reaction

Manuel Molina-Muriel[a,c], Sabrina Campagna Zignani[b], Sara Goberna-Ferrón[a], Antonio Ribera[c], Antonino Salvatore Aricò[b]*, Hermenegildo García[a]*.*

[a]Instituto de Tecnología Química CSIC-UPV, Universitat Politècnica de València and Consejo Superior de Investigaciones Científicas, Universitat Politècnica de València, Av. de los Naranjos s/n, 46022 Valencia, Spain.

[b]Institute of Advanced Energy Technologies (ITAE) of the Italian National Research Council (CNR), Via Salita S. Lucia sopra Contesse 5, 98126 Messina, Italy

[c]Departamento de Química Inorgánica, Universitat de València, Carrer del Doctor Moliner, 50, 46100 Burjassot, Valencia, Spain

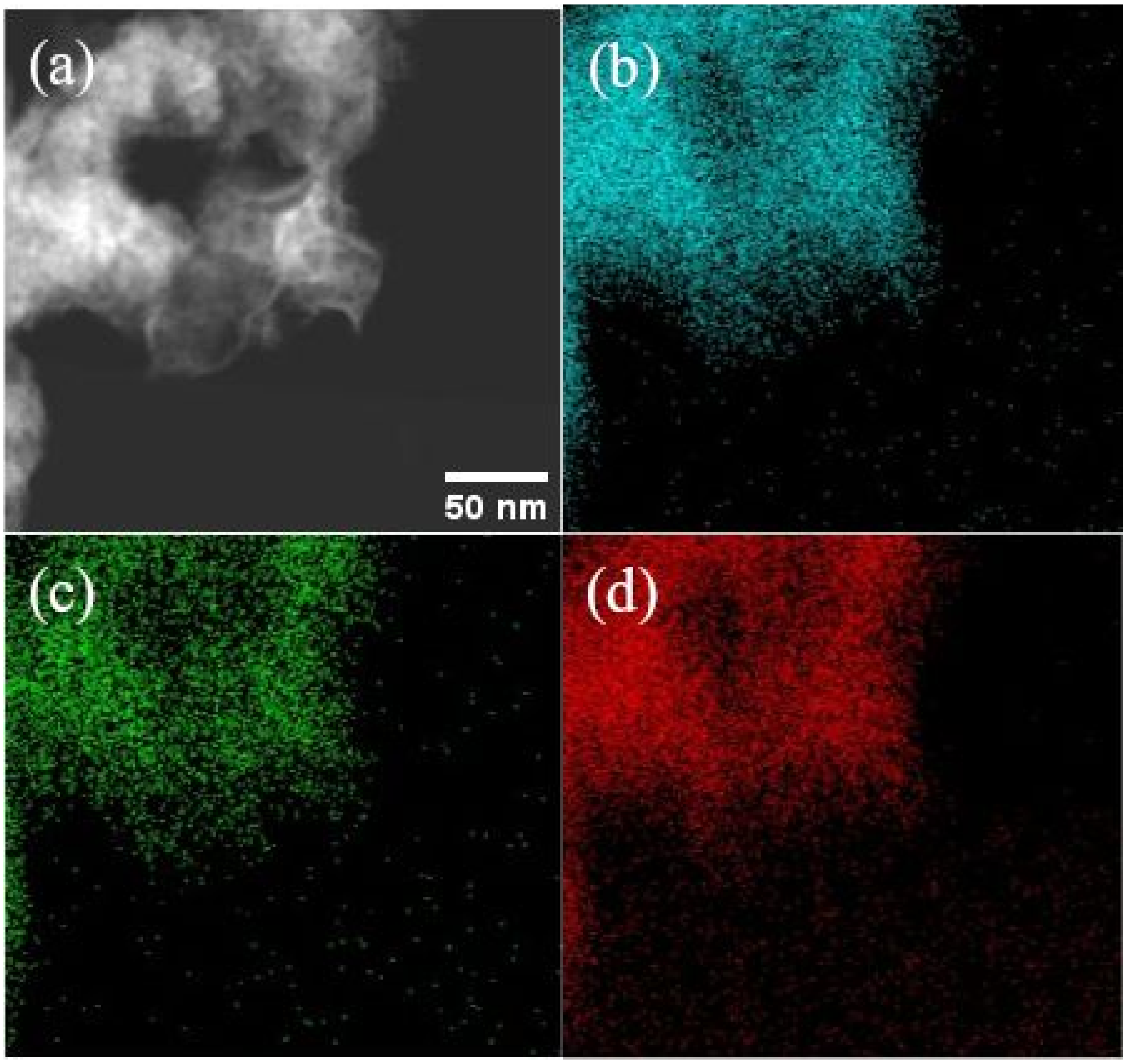

Figure S1. HAADF-STEM image (a) of NiFe-LDH and elemental distribution of Ni (b), Fe (c) and O (d) obtained by EDS.

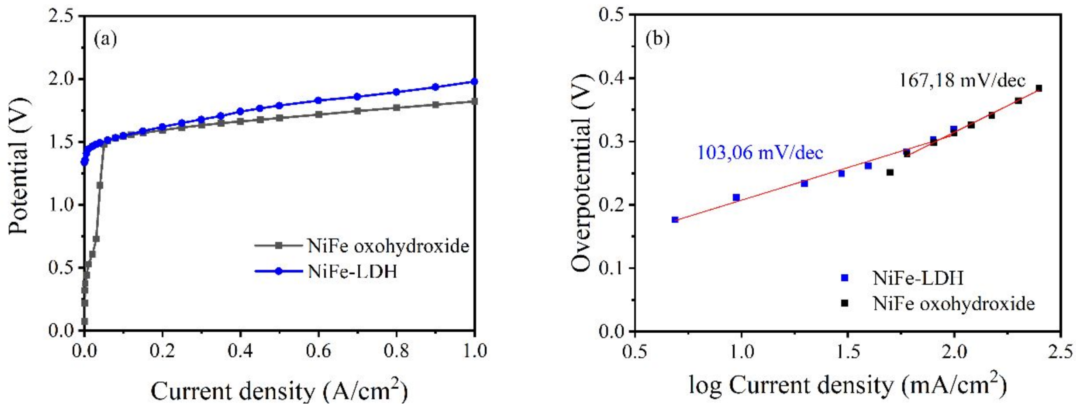

Figure S2. Comparative polarization curves (a) and Tafel slopes (b) for NiFe-LDH and NiFe oxohydroxide reference material.

Table S1. Overpotential and Tafel slope values for NiFe-LDH and NiFe oxohydroxide reference material.

	NiFe-LDH	NiFe oxohydroxide
η at 10 mA·cm^{-2} (mV)	221	313
Tafel slope (mV/dec)	103	167

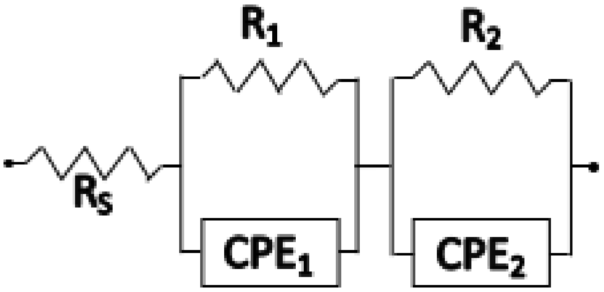

Figure S3. Proposed equivalent circuit model for the electrocatalytic system.

Table S2. Parameters obtained for the equivalent circuit model.

Cell Potential (V)	R_S (mΩ cm^2)	R_1 (mΩ cm^2)	CPE_1		R_2 (mΩ cm^2)	CPE_2	
			Y_1 (S cm^{-2} s^n)	n_1		Y_1 (S cm^{-2} s^n)	n_2
2	289	36.2	52.1 x 10^{-3}	0.708	40.2	4.8	0.714
1.8	263	42.4	26.6 x 10^{-3}	0.529	75.1	1.3	0.766
1.5	283	100.2	114.2 x 10^{-3}	0.616	431.4	0.3	0.811

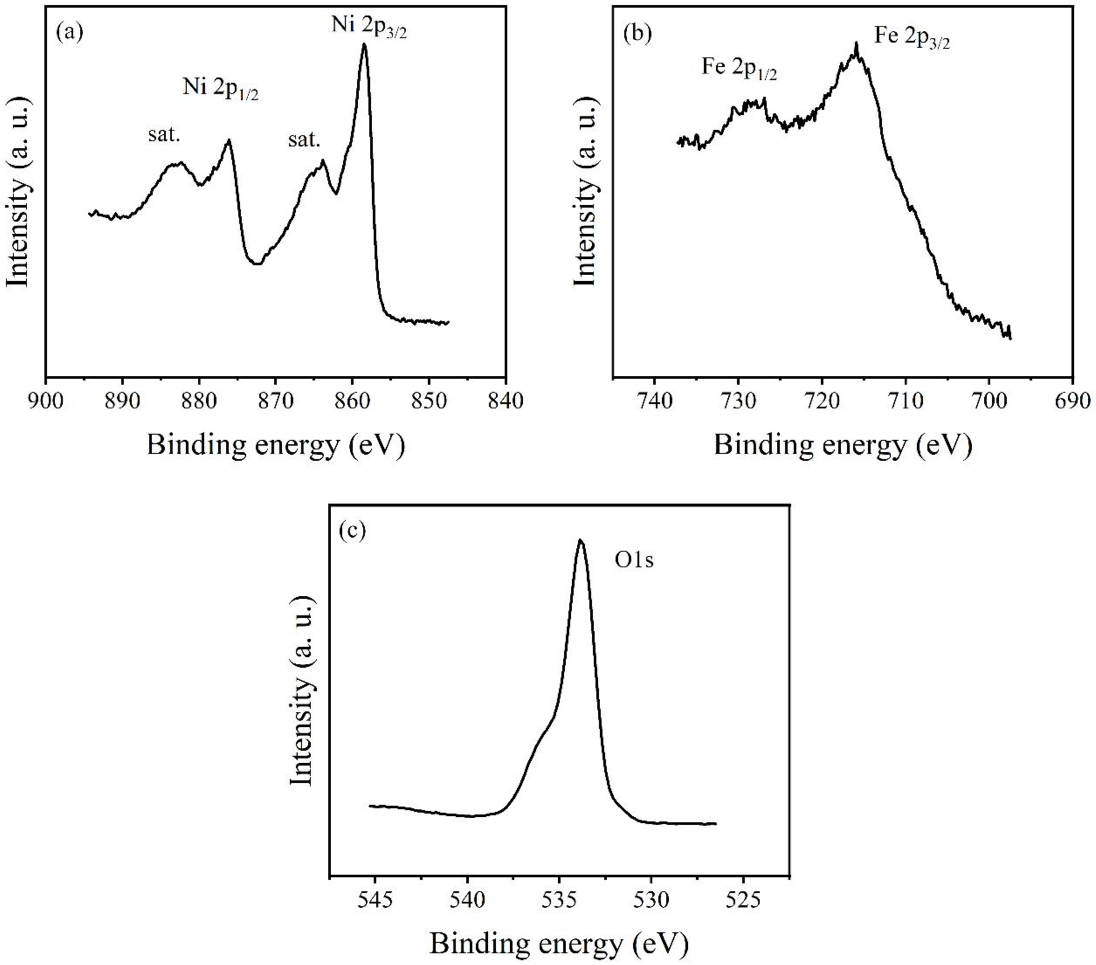

Figure S4. High resolution XPS Ni 2p (a), Fe 2p (b) and O 1s (c) peaks of NiFe-LDH after 35 h durability test.
